# An Optimized Hybrid Deep Learning Model to Detect COVID-19 Misleading Information

**DOI:** 10.1155/2021/9615034

**Published:** 2021-11-15

**Authors:** Bader Alouffi, Abdullah Alharbi, Radhya Sahal, Hager Saleh

**Affiliations:** ^1^Department of Computer Science, College of Computers and Information Technology, Taif University, P. O. Box 11099, Taif 21944, Saudi Arabia; ^2^Department of Information Technology, College of Computers and Information Technology, Taif University, P. O. Box 11099, Taif 21944, Saudi Arabia; ^3^School of Computer Science and Information Technology, University College Cork, Cork, Ireland; ^4^Faculty of Computer Science and Engineering, Hodeidah University, Al Hudaydah, Yemen; ^5^Faculty of Computers and Artificial Intelligence, South Valley University, Hurghada, Egypt

## Abstract

Fake news is challenging to detect due to mixing accurate and inaccurate information from reliable and unreliable sources. Social media is a data source that is not trustworthy all the time, especially in the COVID-19 outbreak. During the COVID-19 epidemic, fake news is widely spread. The best way to deal with this is early detection. Accordingly, in this work, we have proposed a hybrid deep learning model that uses convolutional neural network (CNN) and long short-term memory (LSTM) to detect COVID-19 fake news. The proposed model consists of some layers: an embedding layer, a convolutional layer, a pooling layer, an LSTM layer, a flatten layer, a dense layer, and an output layer. For experimental results, three COVID-19 fake news datasets are used to evaluate six machine learning models, two deep learning models, and our proposed model. The machine learning models are DT, KNN, LR, RF, SVM, and NB, while the deep learning models are CNN and LSTM. Also, four matrices are used to validate the results: accuracy, precision, recall, and *F*1-measure. The conducted experiments show that the proposed model outperforms the six machine learning models and the two deep learning models. Consequently, the proposed system is capable of detecting the fake news of COVID-19 significantly.

## 1. Introduction

A novel coronavirus (COVID-19) was discovered in Wuhan, China, at the beginning of December 2019. The World Health Organization (WHO) has announced that the COVID-19 outbreak is a global pandemic on 11 March 2020 [1]. Due to the panic from COVID-19 disease, people started posting fake news and misinformation about the coronavirus on social media networks. The posts, tweets, and comments contain misleading statements. Recently, the researchers have had a particular interest in utilizing sentiment analysis to distinguish the fake news about COVID-19 [2]. Social media has the biggest contribution for spreading COVID-19 fake news due to the huge number of people's posts having panic expressions. Therefore, the governmental authorities start to launch official websites for COVID-19 announcements to stop circulating fake stories about COVID-19 [3]. Consequently, the researchers began to pay attention to COVID-19 misleading information by analyzing social media contents and applying advanced AI technologies (i.e., machine learning and deep learning) to profiling the COVID-19 fake news [4]. As a result of the research direction in content analysis, the research organizations start raising funding to provide novel solutions to combat COVID-19 in terms of analyzing the misleading information about the COVID-19 pandemic [5–10]. Recently, machine learning and deep learning are playing a vital role in different areas such as sentiment analysis [11, 12]; Alzheimer detection [13]; prediction cancer [14], and others [15, 16]. The researchers have utilized the collected datasets related to COVID-19 through social media to evaluate their proposed approaches [17]. In this work, we have proposed an optimized hybrid model to detect the fake news on COVID-19 on social media. The core idea of the proposed model is the hybridization of using CNN and LSTM.

Our main contributions in this work are as follows:

- Development of a hybrid model integrating CNN and LSTM to detect fake news about COVID-19 is done.
- The proposed model is optimized using a Hyperopt optimization technique to select the optimal values of parameters
- The proposed model, CNN, LSTM, and regular ML algorithms are applied to three COVID-19 fake news datasets
- The experimental results demonstrated that the proposed model had achieved the best performance compared with other models

The rest of this paper is structured as follows.  Section 2 presents the related work.  Section 3 describes the architecture of the proposed system of COVID-19 fake news detection.  Section 4 describes the experimental results. Finally, the paper is concluded in Section 5.

## 2. Related Works

Recently, researchers have been actively working to detect fake news about COVID-19. Wani et al. [18] used CNN, LSTM, and bidirectional encoder representations from transformers (BERT) to detect fake news about COVID-19. They used Contraint@AAAI 2021 Covid-19 Fake news detection dataset [19]. Elhadad et al. [20] proposed a voting ensemble classifier using 10 ML algorithms with seven feature extraction techniques to identify misleading information related to the COVID-19 outbreak. They tested their proposed classifier to 3,047,255 tweets about COVID-19. The best results are obtained from the NN, DT, and LR classifiers. Müller et al. [21] proposed transformer model COVID-Twitter-BERT (CT-BERT), on large a large corpus of Twitter messages about COVID-19. In [22], authors created an annotated dataset about COVID-19 fake news tweets. They proposed a multilingual bidirectional encoder (mBERT) to extract the textual features from the dataset. The results show the mBERT has achieved the highest performance compared with SVM, RF, and a multilayer perceptron. In [17], two stages are developed to detect fake news. The first stage uses a novel fact-checking approach to retrieve the most relevant facts about COVID-19, while the second stage verifies the level of truth by computing the textual entailment. In addition, the authors used pretrained transformer-based language models to retrieve and classify fake news in a particular domain of COVID-19 using BERT and ALBERT. They used a dataset that consists of more than 5000 COVID-19 false claims. In [23], authors gathered COVID-19 news articles from two data sources, and Poynter and Snopes then crawled the sources' textual contents. They classified the articles into 11 various categories. Also, they applied ML algorithms on annotated the articles to detect misinformation about COVID-19. Al-Rakhami et al. [24] proposed an ensemble-learning-based framework to classify the tweets into credible or noncredible. They applied the framework to a large dataset of tweets carrying news about COVID-19. Their framework obtained high accuracy. Hossain et al. [25] introduced a benchmark dataset, COVIDLIES, which contains known COVID-19 misconceptions. They classified each tweet in the dataset into three categories Agree, Disagree, or express No Stance. Patwa et al. [19] created and annotated the dataset that includes 10,700 posts and articles of real and fake news on COVID-19. Four ML baselines: DT, LR, Gradient Boost, and SVM, have been applied to an annotated dataset to classify posts as fake or real. SVM has obtained the best performance with the testing set.

## 3. The Proposed System of Detecting COVID-19 Fake News

**Figure 1:**
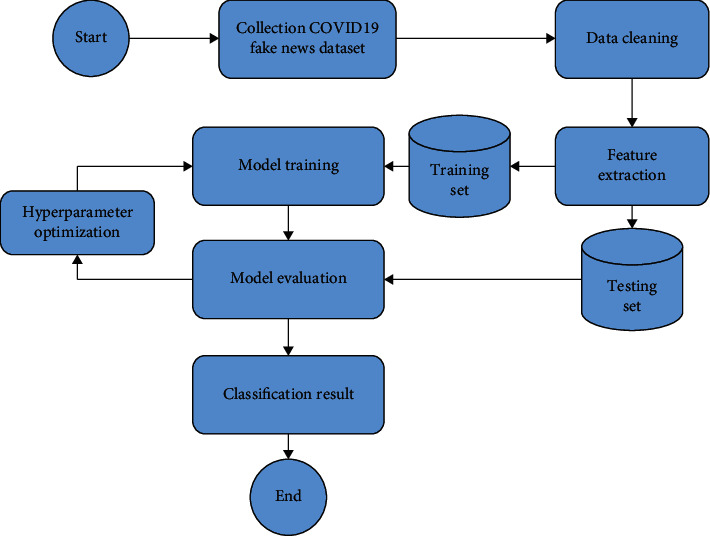
Data fusion steps.

In this section, the proposed system of detecting COVID-19 fake news is introduced.  Figure 1 depicts the workflow of the proposed system showing in a set of steps which are (1) data collection, (2) data cleaning, (3) feature extraction, (4) hyperparameter optimization, and (5) evaluation models.

### 3.1. Data Collection

Three datasets of COVID-19 fake news are used, which are described as follows.

#### 3.1.1. Dataset 1

Dataset 1 was collected from Facebook posts, a far-right website which called Natural News (https://towardsdatascience.com/explore-covid-19-infodemic-2d1ceaae2306).Also, another medicine website is used called orthomolecular.org. Although some data sources are removed from the Internet and social media, they are able to reach by the Internet's Archives.

#### 3.1.2. Dataset 2

Dataset 2 which is COVID-19 fake news data was collected from Internet (https://https://www.researchgate.net/publication/346036811_COVID-19_Fake_News_Data). The dataset 2 consists of a set of COVID-19 fake news. Based on the dataset 2 attributes, the headings used later as labels have the binary attribute. In particular, it has 0, which indicates that the news is fake, while one indicates that the news is true.

#### 3.1.3. Dataset 3

Dataset 3 which is COVID-19 fake news data was collected from Internet (https://www.researchgate.net/publication/349517903_COVID-19_Fake_News_Dataset). Dataset 3 was collected by Webhose.io and then was manually labeled. The dataset consists of three types of news which are (1) false news, which is called fake, (2) true news, and (3) partially false news. For simplification, false news and partially are considered false, labeled as 0, while the real news is labeled as 1.

### 3.2. Data Cleaning

In this phase, we have applied five steps described as follows:

- *Text Parsing*. In this step, the tokenization functions are used to divide the text within the datasets for further analysis.
- *Data Cleaning*. In this step, regular expressions methods are used to extract English alphabets, numbers, and their combination. This step is applied to eliminate any noisy data within the datasets texts.
- *Part of Speech (PoS) Tagging*. This step has marked each word in the text with its root, including a verb, adjective, and noun.
- *Stop Words Removal*. All the common words are removed from the text in this step, such as “a” and “the.”
- *Stemming*. In this step, we have applied a replacement method to replace each word with its root to eliminate the redundancy within the text. In addition, English stemmer is used, which reduces the text by 40–50% concerning the original text within the three datasets.

### 3.3. Feature Extraction Methods

In this phase, two subphases have been done, which are applying Term Frequency-Inverse Document Frequency (TF-IDF) and word embedding for regular ML and DL models, respectively.

- For regular ML models, we have applied the TF-IDF method to assign weights to the prepossessed text of the three datasets [26]. The key idea of the TF-IDF is to determine the word frequencies within the text.
- For DL models, word embedding is a mechanism to represent words into vectors where the words with the same meaning have similar vectors. Thus, every word within the text is represented in dense vectors. Glove is one of the more popular word embedding techniques [27]. Glove is built based on the unsupervised learning technique to generate the word vector representation. At the technical level, we used Glove. 6B.zip word embedding included four dimensions (i.e., 25 d, 50 d, 100 d, and 200 d). According to this work, we have used 200 d vectors to construct our embedding matrix.

### 3.4. The Proposed Model Description

**Figure 2:**
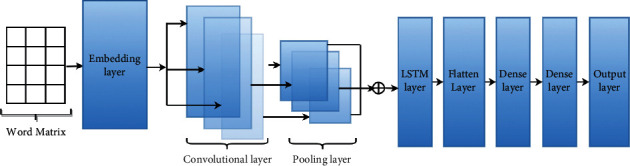
The architecture of the proposed model.

In this section, the architecture of our proposed model is described.  Figure 2 depicts the set of layers of the proposed model, which are sequentially working to detect fake news. In particular, the following layers are used: an embedding layer, a convolutional layer, a pooling layer, an LSTM layer, a flatten layer, a dense layer, and an output layer. In addition, we have applied Hyperopt optimization methods to optimize the proposed model further to select the best values for the proposed model's parameters. The layers are described as follows:

- *Embedding Layer.* Each new has been represented into vectors mapped to each word. At the technical level, we have implemented this layer using Keras library [28]. The Keras library has three parameters which are input-dim, output-dim, and input-length. The input-dim is used to configure the vocabulary size, while the output-dim is used to configure the size of the embedded words. The input-length is used to configure the length of input sequences. According to our implementation, the input-dim, output-dim, and input-length are 20000, 200, and 32, respectively.
- *Dropout Layers.* These layers are used to prevent overfitting and reduce the complexity of the model [29]. As stated earlier, this layer receives its input from the embedding layer output. For configuration, we have set the value of the dropout as range between 0.1 and 0.9.
- *Convolutional Layer.* The convolutional layer receives input from the dropout layer. The convolutional layer has two main parts, which are filter and feature map as a kernel. In the first part, a filter is used to apply filtering on the input word matrix. The filtering process is useful for providing a map of features that indicates the pattern of the input data [30]. The ReLU activation function is used to identify the features within the news.
- *The Pooling Layer*. This layer uses the max operation for feature reduction within the feature mapping process. In particular, configuring a high value will significantly help capture the essential features, which reduces the computation for the next layer.
- *LSTM Layer.* LSTM is a type of recurrent neural network which is used for prediction based on learning long-term dependencies. According to this work, we have used LSTM to build the hybrid model.
- *The Flatten Layer.* The text was converted to a 1-dimensional array by the flatten layer, which was then input to the following layer.
- *Dense Layer*. It is a deeply connected neural network layer. It has some parameters which are input, kernel, bias, and activation. The input parameter represents the input data. The kernel represents the weight data. And the activation is used to represent the activation function.
- *The Output Layer.* This layer is used to take the output of the flattened layers to generate the model's final output, real or fake news. We have used ADAM optimizer [31] and sigmoid activation function [32].

### 3.5. Different Models

Different models have been compared with the proposed model: six regular ML models, CNN, and LSTM.

- Six regular ML models such as DT [33], LR [34], KNN [35], RF [36], SVM [13], and NB [37] were used to compare with proposed model.
- The long short-term memory (LSTM) model has five layers which are (1) an embedding layer, (2) hidden layer, (3) dropout layer, (4) flatten layer, and (5) an output layer. The embedding layer is the first layer that has been designed similarly to the proposed model layers. The hidden layer is used, which is LSTM [38, 39]. In particular, the L2 weight regularization technique is used with reg_rate value for l2. The third layer is the dropout layer which is used to eliminate the overfitting and simplify the model [29]. It is configured by setting the dropout value as a range (0.10.9). The fourth layer is the flattened layer which aims to convert the entire text into a vector of features. Finally, the output layer takes the flatten layer output to generate the final output, which classifies text as the whole in terms of real or fake news.
- Convolutional neural network (CNN) consists of an embedding layer, a convolutional layer, a pooling layer, a flatten layer, a dense layer, and an output layer.

### 3.6. Hyperparameter Optimization

Hyperparameter optimization aims to optimize the hyperparameters for ML and DL models automatically. Hyperparameter tuning is utilized to pass various parameters into the model to select the best values of parameters to achieve the best performance.

- For optimization ML models, we have used grid search. We have defined a set of initial values for each hyperparameter. The model checks these values and then selects the best value for each hyperparameter for obtaining the highest accuracy. Also, K-fold cross-validation (CV) is used where the dataset is equally divided into K-fold. K-1 folds are used for training, and the rest part is used for testing. The dividing process is repeated until the model reaches that each fold has been used for testing. Finally, the classifier is evaluated based on the average of accuracy within the 10-fold.
- We have used Hyperopt, which is distributed asynchronous hyperparameter optimization built-in python library, for optimization of DL algorithms. Furthermore, Hyperopt is an open-source library for large-scale AutoML and HyperOpt-Sklearn (https://github.com/hyperopt/hyperopt). A set of parameters for the proposed model is configured as shown in Table 1. Also for the LSTM model, a set of parameters is as shown in Table 2. Similar to LSTM, a set of parameters of the CNN model is configured as shown in Table 3.

### 3.7. Evaluation Metrics

Four standard machine learning metrics are used, which are accuracy, precision, recall, and *F*1-score. Equations (1)–(4) describe the formulas for calculating these metrics. Accordingly, TP stands for true positive, TN for true negative, FP for false positive, and FN for false negative.

(i) Accuracy is the popular metric used to perform ML and DL models. It measures the percentage of correctly predicted observations. The accuracy calculation formula is as follows:
  (1) $$\begin{array}{c} \text{accuracy}=\frac{\text{TP}+\text{TN}}{\text{TP}+\text{FP}+\text{TN}+\text{FN}}. \end{array}$$
(ii) The second metric is a precision which indicates the ratio of true positives to all true events predicted. The precision calculation formula is as follows:
  (2) $$\begin{array}{c} \text{precision}=\frac{\text{TP}}{\text{TP}+\text{FP}}. \end{array}$$
(iii) Recall is the third metric used to indicate the total number of positive classifications out of true class. The recall calculation formula is as follows:
  (3) $$\begin{array}{c} \text{recall}=\frac{\text{TP}}{\text{TP}+\text{FN}}. \end{array}$$
(iv) The fourth metric is *F*1-score which shows the trade-off between precision and recall. It shows the weighted average of precision and recall. The *F*1-score calculation formula is as follows:
  (4) $$\begin{array}{c} F1-\text{score}=\frac{2\cdot p\text{recision}\cdot \text{recall}}{\text{precision}+\text{recall}}. \end{array}$$

## 4. Results

### 4.1. Experiment Setup

The experiments were conducted using a Google Colab RAM 25 GB, Python 3, and GPU. The three comparable models, the proposed model, LSTM, and CNN models, are implemented using the Keras library. The regular ML models have been implemented using the sci-kit-learn package. For optimization, grid search is used for ML, and the Hyperopt library is used for DL. For the embedding layer, a 200-dimensional word vector is used for the Glove set pretrained. For the datasets, three datasets of COVID-19 fake news are used. Each dataset is divided into 80% for training and 20% for testing. Each experiment has been repeated ten times. The result of cross-validation (CV) and testing performance has been registered.

### 4.2. Results of Dataset 1

#### 4.2.1. Result of Applying ML to Dataset 1

Table 4 describes the obtained performance of CV and the testing validation for ML using dataset 1. Further details are as follows:

- *CV Result*. For DT, bi-gram is registered the best performance (ACC = 79.41%, PRE = 79.83%, REC = 79.09%, and *F*1 = 79.45%), while four-gram has obtained the worst performance (ACC = 67.45%, PRE = 74.49%, REC = 67.63%, and *F*1 = 65.23%). For KNN, unigram is registered the highest performance (ACC = 87.52%, PRE = 88.05%, REC = 87.52%, and *F*1 = 87.46%), while four-gram is registered the worst performance (ACC = 64.83%, PRE = 72.84%, REC = 64.83%, and *F*1 = 61.55%). For LR, unigram is registered the highest performance (ACC = 92.81%, PRE = 92.96%, REC = 92.81%, and *F*1 = 92.81%), while four-gram is registered the worst performance (ACC = 70.48%, PRE = 79.29%, REC = 70.48%, and *F*1 = 67.8%). For RF, unigram is registered the highest performance (ACC = 88.71%, PRE = 89.23%, REC = 88.79%, and *F*1 = 88.86%), while four-gram is registered the worst performance (ACC = 66.92%, PRE = 76.59%, REC = 67.28%, and *F*1 = 63.15%). For SVM, unigram is registered the highest performance (ACC = 92.58%, PRE = 92.71%, REC = 92.58%, and *F*1 = 92.57%), while four-gram is registered the worst performance (ACC = 69.43%, PRE = 79.09%, REC = 69.43%, and *F*1 = 66.39%). For SVM, unigram is registered the highest performance (ACC = 90.77%, PRE = 90.92%, REC = 90.77%, and *F*1 = 90.76%), while four-gram is registered the worst performance (ACC = 72.39%, PRE = 79.16%, REC = 72.39%, and *F*1 = 70.49%).
- *Testing Result.* For DT, bi-gram is registered the highest performance (ACC = 69.27%, PRE = 71.06%, REC = 69.27%, and *F*1 = 69.32%), while four-gram is registered the worst performance (ACC = 58.44%, PRE = 60.02%, REC = 58.44%, and *F*1 = 51.28%). For KNN, unigram is registered the highest performance (ACC = 80.56%, PRE = 81.42%, REC = 80.56%, and *F*1 = 80.4%), while four-gram is registered the worst performance (ACC = 50.05%, PRE = 65.05%, REC = 50.05%, and *F*1 = 34.13%). For LR, unigram is registered the highest performance (ACC = 89.91%, PRE = 89.93%, REC = 89.91%, and *F*1 = 89.91%), while four-gram is registered the worst performance (ACC = 64.55%, PRE = 75.39%, REC = 64.55%, and *F*1 = 60.55%). For RF, unigram is registered the highest performance (ACC = 81.9%, PRE = 82.62%, REC = 81.9%, and *F*1 = 81.82%), while four-gram is registered the worst performance (ACC = 57.62%, PRE = 74.66%, REC = 57.62%, and *F*1 = 49.22%). For SVM, unigram is registered the highest performance (ACC = 89.57%, PRE = 89.57%, REC = 89.57%, and *F*1 = 89.57%), while four-gram is registered the worst performance (ACC = 60.52%, PRE = 61.11%, REC = 60.52%, and *F*1 = 60.16%). For NB, unigram is registered the highest performance (ACC = 88.57%, PRE = 88.75%, REC = 88.57%, and *F*1 = 88.55%), while four-gram is registered the worst performance (ACC = 58.48%, PRE = 60.61%, REC = 58.48%, and *F*1 = 50.6%).

#### 4.2.2. Result of Applying DL to Dataset 1

Table 5 describes the obtained performance of CV and the testing validation for DL using dataset 1. Further details are as follows:

(i) *CV Result*. The proposed model has obtained the best performance (ACC = 99.78%, PRE = 100%, REC = 99.18%, and *F*1 = 99.55%), while LSTM has obtained the lowest performance (ACC = 84.55%, PRE = 83.7%, REC = 84.63%, and *F*1 = 84.69%)
(ii) *Testing Result.* he proposed model has obtained the best performance (ACC = 93.24%, PRE = 92.87%, REC = 93.248%, and *F*1 = 93.02%), while LSTM has obtained the lowest performance (ACC = 78.74%, PRE = 78.56%, REC = 78.66%, and *F*1 = 78.74%)

#### 4.2.3. The Optimum DL Parameter Settings for Dataset 1

The best values of the proposed model's parameters are shown in Table 6. The optimal values for CNN's parameters are shown in Table 7. The optimal settings of the LSTM parameters are shown in Table 8.

### 4.3. Results of Dataset 2

#### 4.3.1. Result of Applying ML to Dataset 2

Table 9 describes the obtained performance of CV and the testing validation for ML using dataset 2. Further details are as follows:

- *CV Result.* For DT, unigram is registered the highest performance (ACC = 96.37%, PRE = 96.0%, REC = 96.38%, and *F*1 = 96.02%), while four-gram is registered the worst performance (ACC = 95.5%, PRE = 94.16%, REC = 95.5%, and *F*1 = 93.44%). For KNN, unigram is registered the highest performance (ACC = 95.94%, PRE = 96.02%, REC = 95.94%, and *F*1 = 94.4%), while four-gram is registered the worst performance (ACC = 95.53%, PRE = 95.03%, REC = 95.53%, and *F*1 = 93.5%). For LR, unigram is registered the highest performance (ACC = 97.27%, PRE = 97.12%, REC = 97.27%, and *F*1 = 96.83%), while four-gram is registered the worst performance (ACC = 95.64%, PRE = 95.55%, REC = 95.64%, and *F*1 = 93.74%). For RF, unigram is registered the highest performance (ACC = 97.27%, PRE = 97.12%, REC = 97.27%, and *F*1 = 96.83%), while four-gram is registered the worst performance (ACC = 95.51%, PRE = 94.31%, REC = 95.49%, and *F*1 = 93.45%). For SVM, unigram is registered the highest performance (ACC = 97.4%, PRE = 96.98%, REC = 97.0%, and *F*1 = 96.33%), while four-gram is registered the worst performance (ACC = 95.57%, PRE = 95.35%, REC = 95.57%, and *F*1 = 93.59%). For NB, unigram is registered the highest performance (ACC = 96.22%, PRE = 95.69%, REC = 96.22%, and *F*1 = 95.81%), while four-gram is registered the worst performance(ACC = 95.00%, PRE = 90.00%, REC = 95.00%, and *F*1 = 93.00%).
- *Testing Result.* For DT, unigram is registered the highest performance (ACC = 95.65%, PRE = 95.16%, REC = 95.65%, and *F*1 = 95.36%), while four-gram is registered the worst performance (ACC = 95.39%, PRE = 94.1%, REC = 95.93%, and *F*1 = 93.29%). For KNN, unigram is registered the highest performance (ACC = 95.44%, PRE = 95.65%, REC = 95.44%, and *F*1 = 93.9%), while four-gram is registered the worst performance (ACC = 95.30%, PRE = 94.2%, REC = 95.30%, and *F*1 = 93.20%). For LR, unigram is registered the highest performance (ACC = 96.69%, PRE = 96.38%, REC = 96.69%, and *F*1 = 96.02%), while four-gram is registered the worst performance (ACC = 95.0%, PRE = 95.0%, REC = 95.0%, and *F*1 = 93.0%). For RF, unigram is registered the highest performance (ACC = 96.22%, PRE = 96.28%, REC = 96.22%, and *F*1 = 94.98%), while four-gram is registered the worst performance (ACC = 95.35%, PRE = 90.91%, REC = 95.35%, and *F*1 = 93.07%). For SVM, unigram is registered the highest performance (ACC = 96.90%, PRE = 96.80%, REC = 96.90%, and *F*1 = 96.04%), while four-gram is registered the worst performance (ACC = 95.39%, PRE = 94.1%, REC = 95.39%, and *F*1 = 93.29%). For NB, unigram is registered the highest performance (ACC = 95.38%, PRE = 94.55%, REC = 95.38%, and *F*1 = 94.85%), while four-gram is registered the worst performance (ACC = 95.02%, PRE = 90.04%, REC = 95.01%, and *F*1 = 93.03%).

#### 4.3.2. Result of Applying DL to Dataset 2

Table 10 describes the obtained performance of CV and the testing validation for DL using dataset 2. Further details are as follows:

- *CV Result.* The proposed model is registered the highest performance (ACC = 99.83%, PRE = 99.1%, REC = 97.47%, and *F*1 = 98.04%), while LSTM is registered the worst performance (ACC = 97.12%, PRE = 77.57%, REC = 90.83%, and *F*1 = 91.29%)
- *Testing Result.* The proposed model is registered the highest performance (ACC = 97.7%, PRE = 97.5%, REC = 97.53%, and *F*1 = 97.7%), while LSTM is registered the worst performance (ACC = 96.84%, PRE = 96.5%, REC = 96.84%, and *F*1 = 96.45%)

#### 4.3.3. The Optimum DL Parameter Settings for Dataset 2

The best values of the proposed model's parameters are shown in Table 11. The optimal values for CNN's parameters are shown in Table 12. The optimal settings of the LSTM parameters are shown in Table 13.

### 4.4. Results of Dataset 3

#### 4.4.1. Result of Applying ML to Dataset 3

Table 14 describes the obtained performance of CV and the testing validation for ML using dataset 3. Further details are as follows:

- *CV Result.* For DT, unigram is registered the highest performance (ACC = 72.21%, PRE = 72.5%, REC = 72.33%, and *F*1 = 72.07%), while four-gram is registered the worst performance (ACC = 67.67%, PRE = 64.84%, REC = 67.59%, and *F*1 = 61.23%). For KNN, unigram is registered the highest performance (ACC = 73.16%, PRE = 76.98%, REC = 73.32%, and *F*1 = 71.69%), while four-gram is registered the worst performance (ACC = 67.44%, PRE = 70.66%, REC = 67.44%, *F*1 = 68.1%). For LR, unigram is registered the highest performance (ACC = 81.71%, PRE = 81.57%, REC = 81.71%, nd *F*1 = 81.06%), while four-gram is registered the worst performance (ACC = 68.87%, PRE = 69.42%, REC = 68.87%, and *F*1 = 61.02%). For RF, unigram is registered the highest performance (ACC = 80.2%, PRE = 79.84%, REC = 79.48%, and *F*1 = 78.21%), while four-gram is registered the worst performance (ACC = 67.41%, PRE = 72.33%, REC = 67.46%, *F*1 = 56.2%). For SVM, unigram is registered the highest performance (ACC = 82.12%, PRE = 82.01%, REC = 82.12%, and *F*1 = 81.63%), while four-gram is registered the worst performance (ACC = 69.1%, PRE = 71.13%, REC = 69.1%, and *F*1 = 60.95%). For NB, bi-gram is registered the highest performance (ACC = 80.25%, PRE = 80.27%, REC = 80.25%, and *F*1 = 80.2%), while four-gram is registered the worst performance (ACC = 69.1%, PRE = 71.16%, REC = 69.23%, and *F*1 = 60.17%).
- *Testing Result*. For DT, unigram is registered the highest performance (ACC = 66.96%, PRE = 66.7%, REC = 66.96%, and *F*1 = 66.81%), while four-gram is registered the worst performance (ACC = 52.98%, PRE = 66.1%, REC = 52.98%, and *F*1 = 52.49%). For KNN, unigram is registered the highest performance (ACC = 69.12%, PRE = 75.72%, REC = 69.12%, and *F*1 = 59.7%), while four-gram is registered the worst performance (ACC = 35.8%, PRE = 67.11%, REC = 35.8%, and *F*1 = 21.7%). For LR, unigram is registered the highest performance (ACC = 74.7%, PRE = 75.14%, REC = 74.7%, and *F*1 = 71.71%), while four-gram is registered the worst performance (ACC = 60.63%, PRE = 69.25%, REC = 60.63%, and *F*1 = 61.4%). For RF, unigram is registered the highest performance (ACC = 75.32%, PRE = 75.98%, REC = 75.32%, and *F*1 = 72.45%), while four-gram is registered the worst performance (ACC = 65.5%, PRE = 58.5%, REC = 65.5%, and *F*1 = 54.0%). For SVM, unigram is registered the highest performance (ACC = 76.92%, PRE = 76.49%, REC = 76.92%, and *F*1 = 75.54%), while four-gram is registered the worst performance (ACC = 65.22%, PRE = 55.61%, REC = 65.22%, and *F*1 = 53.75%). For NB, bi-gram is registered the highest performance (ACC = 72.26%, PRE = 71.11%, REC = 72.26%, and *F*1 = 71.09%), while four-gram is registered the worst performance (ACC = 65.74%, PRE = 58.39%, REC = 65.74%, and *F*1 = 53.86%).

#### 4.4.2. Result of Applying DL to Dataset 3

Table 15 describes the obtained performance of CV and the testing validation for DL using dataset 1. Further details are as follows.

- *CV Result.* The proposed model is registered the highest performance (ACC = 97.7%, PRE = 98.18%, REC = 98.41%, and *F*1 = 98.28%), while LSTM is registered the worst performance (ACC = 88.56%, PRE = 91.19%, REC = 91.89%, and *F*1 = 91.38%)
- *Testing Result.* The proposed model is registered the highest performance (ACC = 77.24%, PRE = 77.87%, REC = 77.24%, and *F*1 = 77.02%), while LSTM is registered the worst performance (ACC = 70.16%, PRE = 69.92%, REC = 70.16%, *F*1 = 69.9%)

#### 4.4.3. The Optimum DL Parameter Settings for Dataset 3

The best values of the proposed model's parameters are shown in Table 16. The optimal values for CNN's parameters are shown in Table 17. The optimal settings of the LSTM parameters are shown in Table 18.

### 4.5. Discussion

The section will be present the best models for each dataset.

#### 4.5.1. The Best Models for Dataset 1

**Figure 3:**
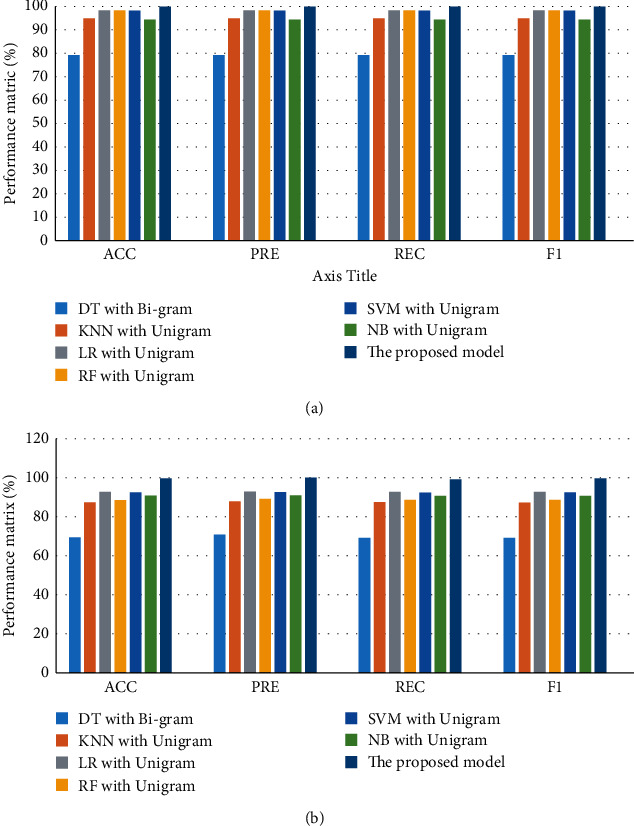
Comparison of performance the best models for dataset 1: (a) CV and (b) testing.

Figure 3 illustrates a comparison between the best models for CV and test performance for dataset 1. For CV performance, the proposed model has the best performance (ACC = 99.78%, PRE = 100%, REC = 99.18%, and *F*1 = 99.55%), while DT with bi-gram has the worst performance (ACC = 69.27%, PRE = 88.05%, REC = 87.52%, and *F*1 = 87.46%). LR with unigram and SVM with unigram have approximately the same performance. For testing performance, the proposed model has the best performance (ACC = 93.24%, PRE = 92.87%, REC = 93.24%, and *F*1 = 93.02%), while DT with bi-gram has the worst performance (ACC = 69.27%, PRE = 71.06%, REC = 69.27%, and *F*1 = 69.32%). LR with unigram and SVM with unigram have approximately the same performance.

Overall, the proposed model has achieved the best performance for CV and testing.

#### 4.5.2. The Best Models for Ddataset 2

**Figure 4:**
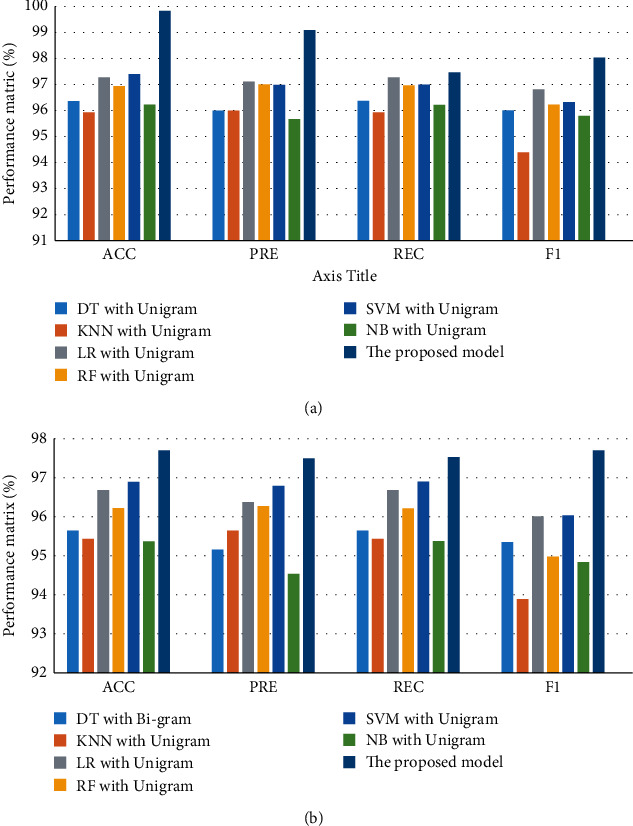
Comparison of performance the best models for dataset 2: (a) CV and (b) testing.

Figure 4 illustrates a comparison between the best models for CV and test performance for dataset 2. For CV performance, the proposed model has the best performance (ACC = 99.83%, PRE = 99.1%, REC = 97.47%, and *F*1 = 98.04%), while KNN with unigram has the worst performance (ACC = 95.94%, PRE = 96.02%, REC = 95.94%, and *F*1 = 94.4%). LR with unigram and SVM with unigram have approximately the same performance. For testing performance, the proposed model has the best performance (ACC = 97.7%, PRE = 97.5%, REC = 97.53%, and *F*1 = 97.7%), while KNN with unigram has the worst performance (ACC = 95.44%, PRE = 95.65%, REC = 95.44%, and *F*1 = 93.9%). LR with unigram and SVM with unigram have approximately the same performance.

Overall, the proposed model has achieved the best performance for CV and testing.

#### 4.5.3. The Best Models for Dataset 3

**Figure 5:**
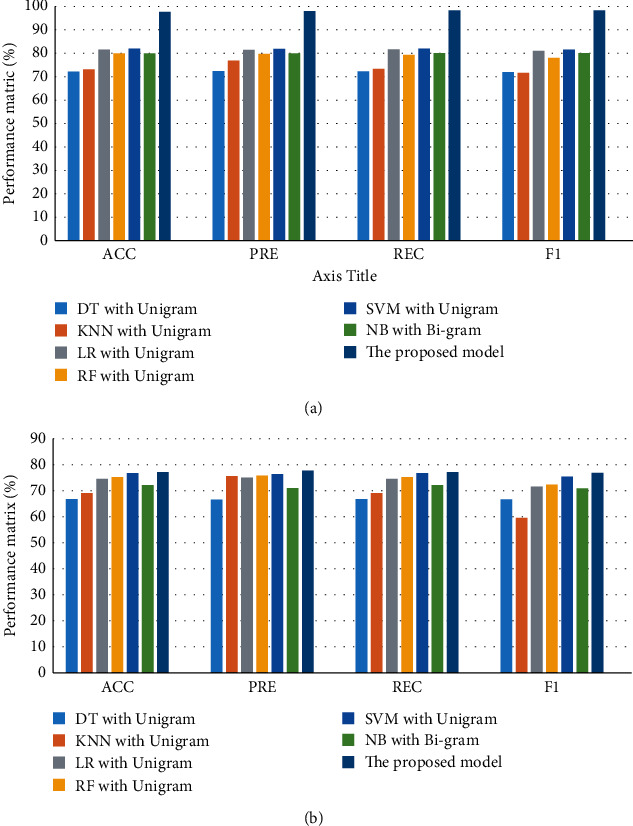
Comparison of performance the best models for dataset 2: (a) CV and (b) testing.

Figure 5 illustrates a comparison between the best models for CV and test performance for dataset 3. For CV performance, the proposed model has the best performance (ACC = 97.7%, PRE = 98.18%, REC = 98.41%, and *F*1 = 98.28%), while DT with unigram has obtained the lowest performance (ACC = 72.21%, PRE = 72.5%, REC = 72.33%, and *F*1 = 72.07%). For testing performance, the proposed model has the best performance (ACC = 77.24%, PRE = 77.87%, REC = 77.24%, and *F*1 = 77.02%), while DT with unigram has the worst performance (ACC = 66.96%, PRE = 66.7%, REC = 66.96%, and *F*1 = 66.81%).

Overall, the proposed model has achieved the best performance for CV and testing.

## 5. Conclusion

In this paper, the hybrid model based on CNN and LSTM has been proposed to detect COVID-19 fake news. Some layers for the proposed model were developed (i.e., an embedding layer, a convolutional layer, a pooling layer, an LSTM layer, a flatten layer, a dense layer, and an output layer. Three datasets about COVID-19 fake news were used to evaluate the proposed model. The experimental results have proved the superiority of our proposed model to detect the fake news of COVID-19 among six machine learning models (i.e., DT, KNN, LR, RF SVM, and NB) and two deep learning models (i.e., CNN and LSTM).

## Figures and Tables

**Table 1:** The parameter values have been adjusted for the proposed model.

Parameters	Values
Filter size	32, 64, 128, 256, 512, 768, 1024
Kernel size	2, 3, 4, 5
Max pooling	3, 6
LSTM unit	Range from 1 to 200
Dense unit 1	Range from 1 to 200
Dense unit 2	Range from 1 to 128
Dropout	0.1, 0.2, 0.3, 0.4, 0.5, 0.6, 0.7, 0.8, 0.9
Batch size	50, 20, 10, 73, 146, 219
Epochs	Rang from 1 to 20

**Table 2:** The parameter values have been adjusted for LSTM.

Parameters	Values
Neurons	Range from 1 to 200
Reg_rate	0.01, 0.05, 0.1, 0.2, 0.3, 0.4, 0.5
Dropout	0.1, 0.2, 0.3, 0.4, 0.5, 0.6, 0.7, 0.8, 0.9
Batch size	50, 20, 10, 73, 146, 219
Epochs	Rang from 1 to 50

**Table 3:** The parameter values have been adjusted for CNN.

Parameters	Values
Filter size	32, 64, 128
Kernel size	2, 3, 4
Max pooling	3, 6
Unit for dense	Range from 1 to 200
Dropout	0.1, 0.2, 0.3, 0.4, 0.5, 0.6, 0.7, 0.8, 0.9
Batch size	50, 20, 10, 73, 146, 219
Epochs	Rang from 1 to 20

**Table 4:** The performance of applying ML to dataset 1

Models	Feature selection methods	Cross-validation performance				Test performance			
		ACC	PRE	REC	*F*1	ACC	PRE	REC	*F*1
DT	Unigram	78.04	78.28	78.11	77.78	68.31	68.57	68.31	68.24
	**Bi-gram**	**79.41**	**79.83**	**79.09**	**79.45**	**69.27**	**71.06**	**69.27**	**69.32**
	Tri-gram	72.04	76.49	72.23	70.86	62.34	69.71	62.34	58.74
	**Four-gram**	**67.45**	**74.49**	**67.63**	**65.23**	**58.44**	**60.02**	**58.44**	**51.28**

KNN	**Unigram**	**87.52**	**88.05**	**87.52**	**87.46**	**80.56**	**81.42**	**80.56**	**80.4**
	Bi-gram	86.7	87.34	86.7	86.62	76.06	76.08	76.06	76.05
	Tri-gram	75.84	78.19	75.84	75.33	54.42	76.31	54.42	42.94
	**Four-gram**	**64.83**	**72.84**	**64.83**	**61.55**	**50.05**	**65.05**	**50.05**	**34.13**

LR	**Unigram**	**92.81**	**92.96**	**92.81**	**92.81**	**89.91**	**89.93**	**89.91**	**89.91**
	Bi-gram	90.7	90.94	90.7	90.68	77.27	82.31	77.27	76.43
	Tri-gram	82.51	84.41	82.51	82.13	69.61	76.45	69.61	67.66
	**Four-gram**	**70.48**	**79.29**	**70.48**	**67.8**	**64.55**	**75.39**	**64.55**	**60.55**

RF	**Unigram**	**88.71**	**89.23**	**88.79**	**88.86**	**81.9**	**82.62**	**81.9**	**81.82**
	Bi-gram	83.05	85.81	83.05	82.32	77.96	78.29	77.96	77.92
	Tri-gram	76.39	79.47	76.55	75.97	64.59	65.27	64.59	64.01
	**Four-gram**	**66.92**	**76.59**	**67.28**	**63.15**	**57.62**	**74.66**	**57.62**	**49.22**

SVM	**Unigram**	**92.58**	**92.71**	**92.58**	**92.57**	**89.57**	**89.57**	**89.57**	**89.57**
	Bi-gram	90.5	90.76	90.5	90.48	86.49	86.52	86.49	86.49
	Tri-gram	80.99	83.52	80.99	80.45	74.93	76.42	74.93	74.63
	**Four-gram**	**69.43**	**79.09**	**69.43**	**66.39**	**60.52**	**61.11**	**60.52**	**60.16**

NB	**Unigram**	**90.77**	**90.92**	**90.77**	**90.76**	**88.57**	**88.75**	**88.57**	**88.55**
	Bi-gram	89.6	90.1	89.6	89.55	82.51	83.5	82.51	82.36
	Tri-gram	78.62	82.79	78.62	77.8	63.07	70.95	63.07	58.96
	**Four-gram**	**72.39**	**79.16**	**72.39**	**70.49**	**58.48**	**60.61**	**58.48**	**50.6**

**Table 5:** The performance of applying DL to dataset 1.

Models	CV performance				Testing performance			
	ACC	PRE	REC	*F*1	ACC	PRE	REC	*F*1
**The proposed model**	**99.78**	**100**	**99.18**	**99.55**	**93.24**	**92.87**	**93.24**	**93.02**
CNN	99.11	95.08	95.39	95.06	78.79	78.88	78.79	78.76
**LSTM**	**84.55**	**83.7**	**84.63**	**84.69**	**78.74**	**78.56**	**78.66**	**78.74**

**Table 6:** The optimum parameter values for dataset 1 for the proposed model.

Parameters	Values
Filter size	768
Kernel size	3
Max pooling	3
LSTM unit	196
Dense unit 1	37
Dense unit 2	106
Dropout LSTM	0.2
Dropout dense 1	0.3
Dropout dense 2	0.4
Batch size	219
Epochs	7

**Table 7:** The optimal CNN parameter values for dataset 1.

Parameters	Values
Filter size	31
Kernel size	2
Max pooling	5
Unit for dense	10
Dropout	0.1
Batch size	73
Epochs	13

**Table 8:** The optimal LSTM parameter values for dataset 1.

Parameters	Values
Neurons	24
Reg rate	0.3
Dropout	0.2
Batch size	73
Epochs	14

**Table 9:** The performance of applying ML to dataset 2.

Models	Feature selection methods	Cross-validation performance				Test performance			
		ACC	PRE	REC	*F*1	ACC	PRE	REC	*F*1
DT	**Unigram**	**96.37**	**96.0**	**96.38**	**96.02**	**95.65**	**95.16**	**95.65**	**95.36**
	Bi-gram	95.86	94.98	95.82	94.66	94.98	93.62	94.98	94.09
	Tri-gram	95.65	94.95	95.62	93.9	95.62	94.68	95.62	93.96
	**Four-gram**	**95.5**	**94.16**	**95.5**	**93.44**	**95.39**	**94.1**	**95.39**	**93.29**

KNN	**Unigram**	**95.94**	**96.02**	**95.94**	**94.4**	**95.44**	**95.65**	**95.44**	**93.9**
	Bi-gram	95.55	95.19	95.55	93.55	95.58	95.2	95.58	93.7
	Tri-gram	95.55	95.19	95.55	93.55	95.64	94.99	95.64	93.9
	**Four-gram**	**95.53**	**95.03**	**95.53**	**93.5**	**95.30**	**94.2**	**95.30**	**93.20**

LR	**Unigram**	**97.27**	**97.12**	**97.27**	**96.83**	**96.69**	**96.38**	**96.69**	**96.02**
	Bi-gram	95.91	95.9	95.91	94.37	95.3	94.29	95.3	94.63
	Tri-gram	95.66	95.71	95.66	93.8	95.57	94.41	95.57	93.94
	**Four-gram**	**95.64**	**95.55**	**95.64**	**93.74**	**95.0**	**94.0**	**95.0**	**93.0**

RF	**Unigram**	**96.95**	**97.01**	**96.97**	**96.24**	**96.22**	**96.28**	**96.22**	**94.98**
	Bi-gram	95.6	95.07	95.59	93.66	95.64	95.78	95.64	93.79
	Tri-gram	95.52	94.56	95.51	93.48	95.49	95.58	95.49	93.43
	**Four-gram**	**95.51**	**94.31**	**95.49**	**93.45**	**95.35**	**90.91**	**95.35**	**93.07**

SVM	**Unigram**	**97.4**	**96.98**	**97.0**	**96.33**	**96.90**	**96.80**	**96.90**	**96.04**
	Bi-gram	97.0	96.80	97.3	96.34	96.78	96.69	96.78	96.02
	Tri-gram	95.73	95.67	95.73	93.97	95.61	94.89	95.61	93.88
	**Four-gram**	**95.57**	**95.35**	**95.57**	**93.59**	**95.39**	**94.1**	**95.39**	**93.29**

NB	**Unigram**	**96.22**	**95.69**	**96.22**	**95.81**	**95.38**	**94.55**	**95.38**	**94.85**
	Bi-gram	95.36	90.93	95.36	93.09	95.35	90.91	95.35	93.07
	Tri-gram	95.36	90.93	95.36	93.09	95.35	90.91	95.35	93.07
	**Four-gram**	**95.00**	**90.00**	**95.00**	**93.00**	**95.02**	**90.04**	**95.01**	**93.03**

**Table 10:** The performance of applying DL to dataset 2.

Models	CV performance				Testing performance			
	ACC	PRE	REC	*F*1	ACC	PRE	REC	*F*1
The proposed model	99.83	99.1	97.47	98.04	97.7	97.5	97.53	97.7
CNN	99.92	92.08	90.83	91.29	97.5	97.32	97.5	97.21
LSTM	97.12	77.57	50.87	58.53	96.84	96.5	96.84	96.45

**Table 11:** The optimum parameter values for dataset 2 for the proposed model.

Parameters	Values
Filter size	1024
Kernel size	2
Max pooling	3
LSTM unit	154
Dense unit 1	53
Dense unit 2	83
Dropout LSTM	0.6
Dropout dense 1	0.2
Dropout dense 2	0.2
Batch size	219
Epochs	15

**Table 12:** The optimal CNN parameter values for dataset 2.

Parameters	Values
Filter size	64
Kernel size	4
Max pooling	6
Unit for dense	28
Dropout	0.4
Batch size	73
Epochs	40

**Table 13:** The optimal LSTM parameter values for dataset 2.

Parameters	Values
Neurons	45
Reg rate	0.05
Dropout	0.5
Batch size	146
Epochs	32

**Table 14:** The performance of applying ML to dataset 3.

Models	Feature selection methods	Cross-validation performance				Test performance			
		ACC	PRE	REC	*F*1	ACC	PRE	REC	*F*1
DT	**Unigram**	**72.21**	**72.5**	**72.33**	**72.07**	**66.96**	**66.7**	**66.96**	**66.81**
	Bi-gram	72.11	72.1	71.91	71.84	64.77	66.18	64.77	65.27
	Tri-gram	68.82	71.98	68.79	69.46	61.14	66.94	61.14	62.14
	**Four-gram**	**67.67**	**64.84**	**67.59**	**61.23**	**52.98**	**66.1**	**52.98**	**52.49**

KNN	**Unigram**	**73.16**	**76.98**	**73.32**	**71.69**	**69.12**	**75.72**	**69.12**	**59.7**
	Bi-gram	70.45	75.12	70.45	62.69	68.17	73.48	68.17	57.92
	Tri-gram	73.32	72.61	73.16	70.95	67.52	69.88	67.52	56.99
	**Four-gram**	**67.44**	**70.66**	**67.44**	**68.1**	**35.8**	**67.11**	**35.8**	**21.7**

LR	**Unigram**	**81.71**	**81.57**	**81.71**	**81.06**	**74.7**	**75.14**	**74.7**	**71.71**
	Bi-gram	77.39	77.87	77.39	75.33	68.75	69.24	68.75	68.96
	Tri-gram	77.28	77.81	77.28	75.12	68.86	69.19	68.86	69.01
	**Four-gram**	**68.87**	**69.42**	**68.87**	**61.02**	**60.63**	**69.25**	**60.63**	**61.4**
RF	**Unigram**	**80.2**	**79.84**	**79.48**	**78.21**	**75.32**	**75.98**	**75.32**	**72.45**
	Bi-gram	73.39	76.34	73.87	68.5	65.71	58.29	65.71	54.0
	Tri-gram	67.82	72.71	67.98	57.21	65.71	58.63	65.71	54.26
	**Four-gram**	**67.41**	**72.33**	**67.46**	**56.2**	**65.5**	**58.5**	**65.5**	**54.0**

SVM	**Unigram**	**82.12**	**82.01**	**82.12**	**81.63**	**76.92**	**76.49**	**76.92**	**75.54**
	Bi-gram	74.71	76.61	74.71	70.95	66.35	62.89	66.35	55.36
	Tri-gram	69.5	71.57	69.5	61.72	66.03	60.97	66.03	55.42
	**Four-gram**	**69.1**	**71.13**	**69.1**	**60.95**	**65.22**	**55.61**	**65.22**	**53.75**

NB	Unigram	79.97	79.89	79.97	78.92	70.62	73.8	70.62	63.58
	**Bi-gram**	**80.25**	**80.27**	**80.25**	**80.2**	**72.26**	**71.11**	**72.26**	**71.09**
	Tri-gram	69.21	71.72	69.21	60.91	66.11	61.87	66.11	54.06
	**Four-gram**	**69.1**	**71.16**	**69.23**	**60.17**	**65.74**	**58.39**	**65.74**	**53.86**

**Table 15:** The performance of applying DL to dataset 3.

Models	CV performance				Testing performance			
	ACC	PRE	REC	*F*1	ACC	PRE	REC	*F*1
**The proposed model**	**97.7**	**98.18**	**98.41**	**98.28**	**77.24**	**77.87**	**77.24**	**77.02**
CNN	93.51	93.92	96.86	95.21	71.79	70.87	71.79	71.08
**LSTM**	**88.56**	**91.19**	**91.89**	**91.38**	**70.16**	**69.92**	**70.16**	**69.98**

**Table 16:** The optimum parameter values for dataset 3 for the proposed model.

Parameters	Values
Filter size	768
Kernel size	3
Max pooling	3
LSTM unit	55
Dense unit 1	127
Dense unit 2	76
Dropout LSTM	0.7
Dropout dense 1	0.6
Dropout dense 2	0.4
Batch size	219
Epochs	10

**Table 17:** The optimal CNN parameter values for dataset 3.

Parameters	Values
Filter size	32
Kernel size	4
Max pooling	3
Unit for dense	6
Dropout	0.4
Batch size	146
Epochs	13

**Table 18:** The optimal LSTM parameter values for dataset 3.

Parameters	Values
Neurons	30
Reg rate	0.01
Dropout	0.4
Batch size	30
Epochs	13

## Data Availability

Three public datasets about COVID-19 fake news are used: (1) https://towardsdatascience.com/explore-covid-19-infodemic-2d1ceaae2306; (2) https://www.researchgate.net/publication/346036811_COVID_Fake_News_Data; and (3) https://www.researchgate.net/publication/349517903_COVID-19_Fake_News_Dataset.
